# Similar Neural Correlates of Planning and Execution to Inhibit Continuing Actions

**DOI:** 10.3389/fnins.2018.00951

**Published:** 2018-12-12

**Authors:** Kei Omata, Shigeru Ito, Youhei Takata, Yasuomi Ouchi

**Affiliations:** ^1^Department of Biofunctional Imaging, Medical Photonics Research Center, Hamamatsu University School of Medicine, Hamamatsu, Japan; ^2^Hamamatsu Medical Photonics Foundation, Hamamatsu PET Imaging Center, Hamamatsu, Japan; ^3^Hamamatsu Photonics KK, Global Strategic Challenge Center, Hamamatsu, Japan

**Keywords:** behavioral inhibition, simple finger tapping, voluntary decision, cued judgment, neural substrates, functional magnetic resonance imaging

## Abstract

Inhibition of action is involved in stopping a movement, as well as terminating unnecessary movement during performance of a behavior. The inhibition of single actions, known as response inhibition (Inhibition of the urge to respond before or after actions) has been widely investigated using the go/no-go task and stop signal task. However, few studies focused on phase and volition-related inhibition after an action has been initiated. Here, we used functional magnetic resonance imaging (fMRI) to investigate the neural correlates of planning and execution underlying the voluntary inhibition of ongoing action. We collected fMRI data while participants performed a continuous finger-tapping task involving voluntary and involuntary (externally directed) inhibition, and during the initiation of movement. The results revealed areas of significantly greater activation during the preparation of inhibition of an ongoing action during voluntary inhibition, compared with involuntary inhibition, in the supplementary (SMA) and pre-supplementary motor areas, dorsolateral prefrontal cortex, inferior frontal gyrus (IFG), inferior parietal lobe, bilateral globus pallidus/putamen, bilateral insula and premotor cortex. Focusing on the period of execution of inhibition of ongoing actions, an event-related fMRI analysis revealed significant activation in the SMA, middle cingulate cortex, bilateral insula, right IFG and inferior parietal cortex. Additional comparative analyses suggested that brain activation while participants were planning to inhibit an ongoing action was similar to that during planning to start an action, indicating that the same neural substrates of motor planning may be recruited even when an action is ongoing. The present finding that brain activation associated with inhibiting ongoing actions was compatible with that seen in response inhibition (urge to stop before/after actions) suggests that common inhibitory mechanisms for motor movement are involved in both actual and planned motor action, which makes our behavior keep going seamlessly.

## Introduction

Inhibition plays an important role in daily life. For example, inhibition of potentially harmful behaviors such as over-eating and over-drinking, and limiting the time spent playing computer games or browsing the web, are important for maintaining physical and mental health. Inhibition of motor actions has been investigated with the “response inhibition” paradigm using go/no-go tasks and stop signal tasks in which a participant is required to inhibit a planned and/or already initiated action (Sasaki et al., 1989; Liddle et al., 2001; Aron et al., 2004; Brass and Haggard, 2007; Lee et al., 2016). Response inhibition is the ability to hold one's own response to distractions which may disturb maintaining attention to the present task. Previous studies of the neural correlates of response inhibition have identified the involvement of the pre-supplementary motor area (pre-SMA), supplementary motor area (SMA), pre-motor cortex, parietal cortex, inferior frontal gyrus (IFG), insular cortex, dorsolateral prefrontal cortex (DLPFC), anterior cingulate cortex (ACC), putamen and globus pallidus (Menon et al., 2001; Garavan et al., 2006; Picton et al., 2007; Simmonds et al., 2008; Bari and Robbins, 2013; Schel et al., 2014). Similarly, the inhibition of ongoing actions like talking, walking and driving are necessary in daily life. However, the neural mechanism of inhibition of ongoing actions remains unclear.

Motor action involves a combination of various processes in the motor control regions, including the premotor cortex, pre-SMA, SMA, and primary motor cortex (Donoghue and Sanes, 1994; Haggard, 2008), and voluntary action involves a set of purposeful processes prior to initiating action (Deecke, 1996; Cunnington et al., 2002, 2003; Haggard, 2008). Based on previous findings, we assumed that the process of inhibition of ongoing actions could be dissociated into two phases: planning and execution of the inhibition of ongoing actions. The planning phase of motor actions has been examined in the context of preparation of motor movements and intention (Libet et al., 1983; Haggard, 2008; Soon et al., 2008; Guggisberg et al., 2011; Guggisberg and Mottaz, 2013). In general, changes in cortical activities that precede an action are considered to reflect processes associated with the planning of a motor action (Guggisberg and Mottaz, 2013). Therefore, we attempted to clarify which brain regions are related to the planning and execution of inhibition of ongoing actions by segmenting the time domain into the period before and just after execution of inhibition. Moreover, previous studies have explored voluntary movement by comparing task conditions in which subjects executed actions that were self-paced or stimulus-triggered (Jahanshahi et al., 1995; Rizzolatti et al., 1998; Jenkins et al., 2000; Cunnington et al., 2002; Haggard, 2008; Shibasaki, 2012). In the present study, we examined the voluntary process of inhibition of ongoing actions, focusing on self-paced and stimulus-triggered conditions. In addition, we examined the planning state with and without actions, and explored the neural substrates for the planning of initiation of ongoing actions, compared with their inhibitory counterparts.

Previous studies have reported that the planning phase of a motor action involves activation in the SMA and pre-SMA (Cunnington et al., 2005; Shibasaki, 2012), the premotor cortex (Weinrich and Wise, 1982; Weinrich et al., 1984) and the inferior parietal lobe (Desmurget et al., 2009; Teixeira et al., 2014). Furthermore, it has been suggested that the mechanism of inhibition of ongoing actions in a cursor tracking task might be shared with that of response inhibition (Morein-Zamir et al., 2004). Therefore, we hypothesized that (i) these brain regions may be associated with the planning phase during ongoing actions, and (ii) the neural correlates of the execution phase of the inhibition of ongoing actions may be similar to those of response inhibition. To clarify these hypotheses about the planning and execution of voluntary inhibition of ongoing action, we investigated the neural correlates of inhibition of an ongoing action using functional magnetic resonance imaging (fMRI) while participants performed a novel finger-tapping task that includes self-paced or stimulus-triggered inhibition of ongoing finger-tapping. For comparison, we also measured brain activity during the initiation of an ongoing action.

## Materials and Methods

### Participants

Twenty-six healthy volunteers (12 females, mean age: 23.4 ± 4.6 years) participated in the present study. The ethics committee of the Hamamatsu University School of Medicine approved the study, and all subjects gave written informed consent. According to the approved procedure, subjects with a current or previous history of neurological or psychiatric disorders and those with metal implants were excluded from the study. Using the Edinburgh handedness inventory (Oldfield, 1971), we confirmed that all subjects but one were right handed. All subjects had normal or corrected-to-normal vision, and normal finger movement.

### Instruments (fMRI Data Acquisition)

MRI scans were conducted with a 3-Tesla scanner (Ingenia; Royal Philips, Eindhoven, Nederland). A 15-channel transmitter-receiver coil was used to scan the head. The T1-weighted sequence (Turbo field echo) was used for anatomic referencing of the fMRI recording, coregistration and normalization [TR, shortest (6 ms); TE, shortest (2.7 ms); flip angle, 8°; voxel size, 0.9 × 0.9 × 0.9 mm; 210 slices]. For functional scans, T2^*^-weighted, gradient-echo, echoplanar imaging was used (TR, ,2500 ms; TE, 25 ms; flip angle, 90°; voxel size, 3 × 3 × 3 mm; 46 interleaved transversal slices). Slice orientation was tilted −20° from the AC-PC line. Two hundred image volumes were acquired in each session, and the first two volumes were discarded to avoid magnetic saturation effects. The total time per session was 8 min and 27 s. To prevent head motion, each subject's head was tightly fixed using cushions. Subjects were instructed to keep their head and body as still as possible during MRI scanning, except their right index finger.

An MRI-compatible head mounted display (Visual Stim Digital; Resonance Technology Company, Inc., Los Angeles, USA) and Presentation software (Neurobehavioral Systems, Inc., San Francisco, USA) on a PC (Windows 7, Dell PRECISION T5500) were used to control stimulus presentation. Visual stimuli were presented via the head mounted display (with 800 x 600 pixel resolution, 30° of horizontal and 22.5° of vertical field of view). The size of the letters and the plus sign were equal to 2.25° of horizontal and 3° of vertical field of view, respectively. For some subjects, a pair of glasses was attached to the head mounted display to correct their vision. A keypad with four buttons and a computer interface (HHSC-1 × 4-L and FIU-904; Current Designs, Inc., Philadelphia, USA) was used for recording the finger-tapping responses. During the experiment, subjects wore headphones that attenuated the fMRI scanning noise, and remained lying down on the scanner bed with a button pad and an emergency buzzer.

### Task Design

A new finger-tapping task was designed to investigate brain activity during behavioral change, and during the planning stages before behavioral change. Subjects were asked to continuously tap a button on a keypad with the index finger of their right hand, under voluntary and involuntary conditions. To clarify the volition of inhibition of ongoing actions, we compared the brain activation between the voluntary and forced inhibition of ongoing actions. In addition, the brain state during the initiation of ongoing actions was also recorded to compare with that of inhibition. There were four conditions in total (voluntary and forced, stop and start of finger-tapping), presented in short blocks lasting 20 s. Each condition appeared six times per session, in a random order. Three sessions were conducted per subject. In total, each condition was presented 18 times for each subject. In all conditions, a white plus sign was presented as a fixation point.

**Figure 1:**
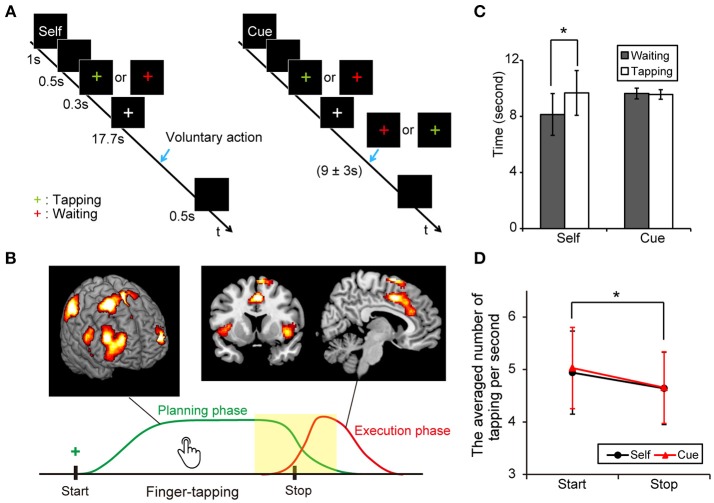
Experimental protocol and behavioral results. **(A)** Illustration of voluntary and forced conditions of start and stop of an ongoing finger tapping. A blue arrow indicates the occurrence of a behavioral shift. **(B)** A scheme of the voluntary inhibition of ongoing actions. Subjects are requested to start a continuous finger tapping for a while and stop it by themselves if a green plus sign is presented. Green and red lines indicate presumed curves of brain activation during the planning and execution phases, respectively. A center of interest in this study was the difference in brain activities around the behavioral change (yellow). **(C)** Mean times for the periods from the presentation of a cue (green or red plus) to the execution of behavioral shifts in each condition (Error-bar: *SD*, paired *t*-test; *p < 0.001). **(D)** Averaged numbers of tapping per second in each condition (Error-bar: *SD*, *p < 0.05).

Figure 1A shows the four task conditions. In the “self” condition, subjects were instructed to start or stop finger-tapping voluntarily. An instruction cue, “self,” was provided for 1 s at the start of a trial block. After a blank screen (black background) appeared for 0.5 s, a green- or red-colored plus sign was presented for 0.3 s as a cue to start finger-tapping, or wait to begin finger-tapping, respectively. Participants were then instructed to start and stop finger-tapping voluntarily during the following 17.7 s. Thus, the participants should stop or start a finger tapping within the block depending on the volunteer's judgment when “self” cue was presented at the start point. We instructed them to keep a tapping or a waiting for a while and then take a proper action whenever they wanted. At the end of each trial block, a blank screen was presented for 0.5 s. Then, participants were instructed to stop tapping as quickly as possible. In the “cue” condition, the task was similar to the “self” condition except that subjects were cued to start or stop finger-tapping. A green- or red-colored plus sign appeared for 0.3 s to cue participants to start or stop finger-tapping, respectively, during the 17.7 s period. A color change occurred at 9 s, with an equal probabilistic variation of ± 3 s after cue onset. The timing of the cue was based on the findings of a pilot study.

Subjects practiced the tasks for several minutes outside the scanner before MRI scanning, and were instructed to tap a button at a natural speed. However, some participants occasionally failed to produce an action on some trials in the voluntary condition because they were asked to keep tapping or waiting, based on their own judgment. Trials involving an extremely short duration of action (< 1 s), no action, or an extremely long duration of action (>17 s) were excluded from subsequent analysis. Additionally, the performance criterion for each subject included a success rate of more than 90% in all conditions. Three subjects were excluded from the final analysis because of low task performance.

### Behavioral Analysis

To examine each subject's performance, behavioral data from the tasks were analyzed, calculating the average duration of tapping and the average number of taps per second in the period between the presentation of the colored plus sign and the time of execution of behavioral changes. The data from error trials were excluded from this analysis. The timing of execution was defined by the first button-press at the start of the ongoing tapping or the last button-press when the ongoing tapping stopped. Note that the presentation time of the stop or start cue in the forced conditions was not used for determining the timing of execution of behavioral changes to achieve equality between voluntary and forced conditions. These times were used for further fMRI analysis.

### fMRI Analysis

fMRI data were analyzed using SPM8 (http://www.fil.ion.ucl.ac.uk/spm/) in MATLAB (MathWorks, Natick, MA, USA). Preprocessing of the fMRI was as follows: slice timing correction, realignment, coregistration, spatial normalization to the standard Montreal Neurological Institute (MNI) template, and spatial smoothing using a Gaussian kernel with a full width at half-maximum of 8 mm.

For each subject, the brain regions were statistically evaluated using a general linear model (GLM) including both explanatory variables of interest and variables of non-interest, and six realignment parameters were used as multiple regressors. To clarify brain activation during the planning and execution phases (Figure 1B), the model included the following four event-related regressors: the timing of inhibition and initiation of finger-tapping in both the voluntary and forced conditions and eight box regressors: waiting period after stopping finger-tapping, waiting period before starting finger-tapping, tapping period before stopping finger-tapping and tapping period after starting finger-tapping in both the voluntary and forced conditions. These regressors were convolved with a hemodynamic-response function. Bain activity reflecting the neural processes underlying the planning of voluntary action in terms of when to stop or start finger-tapping was detected using two contrast values (i.e., voluntary minus forced): (*i*) contrast of tapping periods before stopping finger-tapping between voluntary and forced conditions, and (*ii*) contrast of waiting periods before starting finger-tapping between voluntary and forced conditions. Note that the contrast (*i*) and (*ii*) reflect activation of “planning” because subjects were required to decide when to stop or start in the voluntary conditions whereas just waiting in the forced conditions. Especially, the activations related to tapping action itself in both the voluntary and forced conditions were canceled out in the contrast (*i*). Brain activity related to the execution of voluntary inhibitory action was detected using two contrast values, as above: (*i*) contrast of execution of inhibition of ongoing actions between the voluntary and forced conditions, and (*ii*) contrast of initiation of ongoing actions between the voluntary and forced conditions. Because the execution of motor actions must be correlated with a physical movement, the event-related regressors of the timing of an inhibitory or initiatory action were assumed to reflect the neural process of the execution of inhibition of ongoing actions.

At the first level, the contrast images corresponding to the regressors were calculated for each subject using a GLM. We then conducted a second-level analysis of the event-related contrasts and the voluntary/ forced contrasts. One-sample *t*-tests for each contrast were conducted to verify the relevant brain regions. For all data, a threshold of uncorrected *P* < 0.001 for peak-level and a cluster-level family-wise error (FWE) of 0.05 was used for statistical analyses. In addition, two conjunction analyses were performed to confirm the common brain regions related to inhibition of finger-tapping (both voluntary and forced stopping) and planning when to stop or start finger-tapping. A threshold of uncorrected *P* < 0.001 for peak-level and a cluster-level FWE of 0.05 was also used. Atlases of the human brain (Anatomy Toolbox; Eickhoff et al., 2007) in SPM8 and the Automated Anatomical Labeling map (Tzourio-Mazoyer et al., 2002) in MRIcro were used for anatomical references.

### Data Availability

Anonymized data not published within the article will be shared on reasonable request from any qualified investigator.

## Results

### Behavioral Results

The average time durations (sec) between the presentation of the colored plus sign and the start of behavioral change were calculated in the voluntary start condition (*M* = 8.13, *SD* = 1.49, range = 5.57–11.93), the voluntary stop condition (*M* = 9.63, *SD* = 1.60, range = 6.94–13.14), the forced start condition (*M* = 9.67, *SD* = 0.38, range = 8.95–10.73) and the forced stop condition (*M* = 9.56, *SD* = 0.34, range = 9.00–9.97). Our data showed that the average duration between the sign of the task cue and the start point of motor movements were significantly shorter in the voluntary start condition than in the voluntary stop condition (paired *t*-test: *t*_22_ = 5.89, *P* < 0.001). Conversely, there was no difference between the forced conditions (paired *t*-test: *t*_22_ = 1.08, *P* = 0.29) (Figure 1C). These results indicate that subjects started finger-tapping earlier in the voluntary start condition than the voluntary stop condition, and responded to the cue signals appropriately.

A two-way repeated measures ANOVA was performed on the number of taps per second with active-passive (voluntary or forced action) and behavioral change (start or stop condition) as factors. The main effect of behavioral shift was significant [*F*_(1, 88)_ = 4.60, *P* = 0.035], indicating that subjects tapped a button faster in the start conditions than in the stop conditions. In contrast, neither the main effect of active-passive [*F*_(1, 88_ = 0.11, *P* = 0.74] nor the interaction of the two factors [*F*_(1, 88_ = 0.06, *P* = 0.81] was significant (Figure 1D).

### Neuroimaging Results

**Figure 2:**
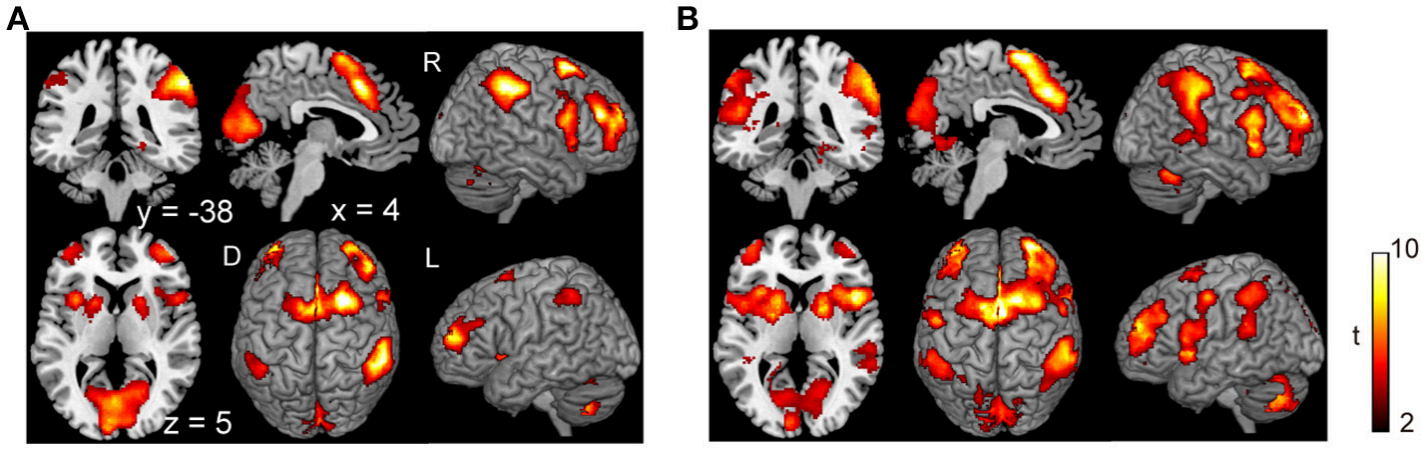
Comparison of the brain activity before the execution of inhibition between voluntary and forced conditions. Activations in the planning phase of an ongoing action task while tapping **(A)** and while waiting **(B)** are shown. The color on the brain images indicates the significant difference between the voluntary and forced conditions (self-initiated > stimuli-triggered). Thresholds of activation height and extent were set at the *P* < 0.001 level (uncorrected) and a cluster-level FWE of 0.05, respectively. The scale for t-scores is shown alongside. The number and alphabetic characters indicate the XYZ coordinates in the Montreal Neurological Institute (MNI) space. The letters in the figure indicate the direction of each brain image (L, left; R, right; D, dorsal).

Subjects were asked to decide when to execute an inhibition of continuous finger tapping or of initiation of the tapping behavior in a voluntary manner. The contrast of brain activity during tapping or waiting before action execution between the voluntary and forced conditions represents a brain network involved in planning and executing motor actions as follows; during the taping period, the contrast of voluntary with forced inhibition of continuous finger-tapping revealed peak activations in the bilateral DLPFC, the bilateral IPL, the bilateral cerebellum, the primary visual cortex, the right IFG (Brodmann area [BA] 44), the left insula and the right globus pallidus/putamen (Figure 2A and Table 1). In addition, during the waiting period, the contrast of voluntary with forced initiation of continuous tapping revealed peak activations in the left SMA, the right IFG, the left globus pallidus/putamen, the left cerebellum, the left DLPFC, the right IPL and the left angular gyrus (Figure 2B and Table 2). Despite these different behavioral states, large parts of areas of activation between planning while tapping and planning while waiting were overlapped, regardless of whether finger-tapping was happening, as confirmed by a conjunction analysis (Figure 5A and Table 5).

**Table 1:** Local maxima for the contrast of the tapping period before voluntary against forced stop of finger-tapping.

**Regions**	**Z-scores**	**MNI coordinates, x, y, z**			**Number of voxels**
Right SMA	6.67	26	12	58	4674
Right dorsolateral prefrontal cortex	6.55	42	40	22	1726
Right inferior parietal lobe	6.28	54	−40	46	2012
Left cerebellum	5.96	−30	−62	−30	1236
Left dorsolateral prefrontal cortex	5.72	−32	58	14	1210
Visual cortex (V1)	5.63	−8	−90	2	5075
Right inferior frontal gyrus (BA44)	5.53	56	8	18	1242
Left insula, globus pallidus	5.41	−34	12	2	703
Right cerebellum	5.07	32	−60	−26	730
Left inferior parietal lobe	4.39	−46	−48	50	560
Right globus pallidus/putamen	4.35	20	12	6	354

*All clusters survived the peak threshold of p < 0.001 level (uncorrected) and a cluster-level FWE of 0.05. SMA, supplementary motor area; BA, Brodmann area*.

**Table 2:** Local maxima for the contrast of the waiting period before voluntary against forced start of finger-tapping.

**Regions**	**Z-scores**	**MNI coordinates, x, y, z**			**Number of voxels**
Left SMA	7.14	−8	2	66	10528
Right inferior frontal gyrus,globus pallidus	6.18	46	12	6	3010
Left globus pallidus/putamen	5.92	−16	4	10	3428
Left cerebellum	5.77	−40	−60	−32	11445
Left dorsolateral prefrontal cortex	5.65	−38	52	22	2089
Right inferior parietal lobe	5.57	56	−36	44	3065
Left angular gyrus	4.82	−50	−38	22	2114

*All clusters survived the peak threshold of p < 0.001 level (uncorrected) and a cluster-level FWE of 0.05. SMA, supplementary motor area*.

**Figure 3:**
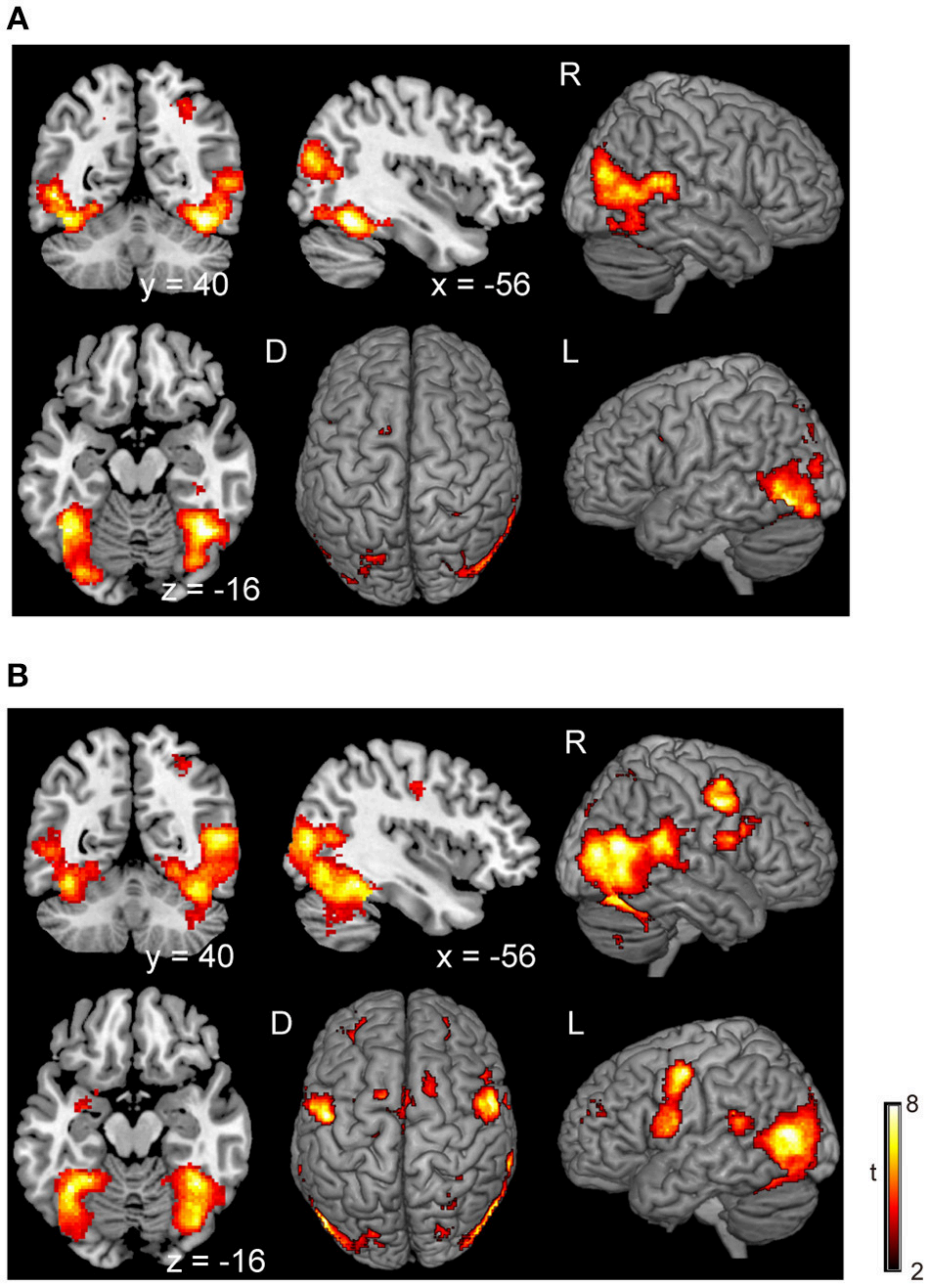
Comparison of the brain activity between forced and voluntary conditions in the stop and start conditions. The color on the brain images indicates the significant difference between the forced and voluntary conditions (stimuli-triggered > self-paced). **(A)** Stop condition, **(B)** start condition. The height and extent thresholds of brain activation were set at the *P* < 0.001 level (uncorrected) and a cluster-level FWE of 0.05, respectively. The scale for t-scores is shown alongside. The number and alphabetic characters indicate the XYZ coordinates in the Montreal Neurological Institute (MNI) space. The letters in the figure indicate the direction of each brain image (L, left; R, right; D, dorsal).

There was no significant difference in activation during the execution of voluntary stop compared with the execution of forced stop (self-paced > stimulus-triggered). In contrast, brain activation in the occipital regions (BA19/37), which include the fusiform gyrus and middle occipital gyrus, were found in the comparison between forced and voluntary stop (stimulus-triggered > self-paced) (Figure 3A and Table 3), suggesting greater visual load in the stimulus-triggered condition. A similar pattern was observed in the initiation conditions. And then, we found additional activation of the premotor cortex and globus pallidus/putamen in the comparison between forced vs. voluntary start (Figure 3B and Table 3), suggesting that forced start requires control from the basal ganglia.

**Table 3:** Local maxima for the contrast of the forced against voluntary action in the execution of inhibition and initiation.

	**Regions**	**Z-scores**	**MNI coordinate, x, y, z**			**Number of voxels**
Inhibition	Right fusiform gyrus, inferior occipital gyrus, middle occipital gyrus	5.81	40	−56	−16	5095
	Left inferior occipital gyrus, inferior temporal gyrus, fusiform gyrus	5.80	−44	−70	−12	2645
	Left superior occipital gyrus	4.73	−24	−70	44	651
	Left SMA	4.60	−10	6	58	172
	Left premotor cortex	4.16	−44	4	34	187
Initiation	Left Premotor cortex	5.78	−50	−8	48	891
	Right Middle temporal gyrus, fusiform gyrus, middle occipital gyrus	5.78	48	−64	14	6344
	Left SMA	5.66	−8	2	62	1241
	Left Middle occipital gyrus, fusiform gyrus, middle temporal gyrus	5.63	−50	−76	8	4400
	Right Premotor cortex	5.47	54	0	40	538
	Left pallidus/ putamen	5.45	−18	0	4	1393
	Left middle frontal gyrus	4.88	−24	48	28	451
	Right Middle cingulate cortex	4.77	14	−16	38	234
	Right middle frontal gyrus	4.64	22	48	26	166
	Right inferior frontal gyrus	4.59	64	6	18	381
	Right pallidus	4.54	20	8	2	405
	Left Middle cingulate cortex	4.44	−12	10	32	242
	Right superior parietal lobe	3.80	34	−52	54	127

*All clusters survived the peak threshold of p < 0.001 level (uncorrected) and a cluster-level FWE of 0.05*.

**Figure 4:**
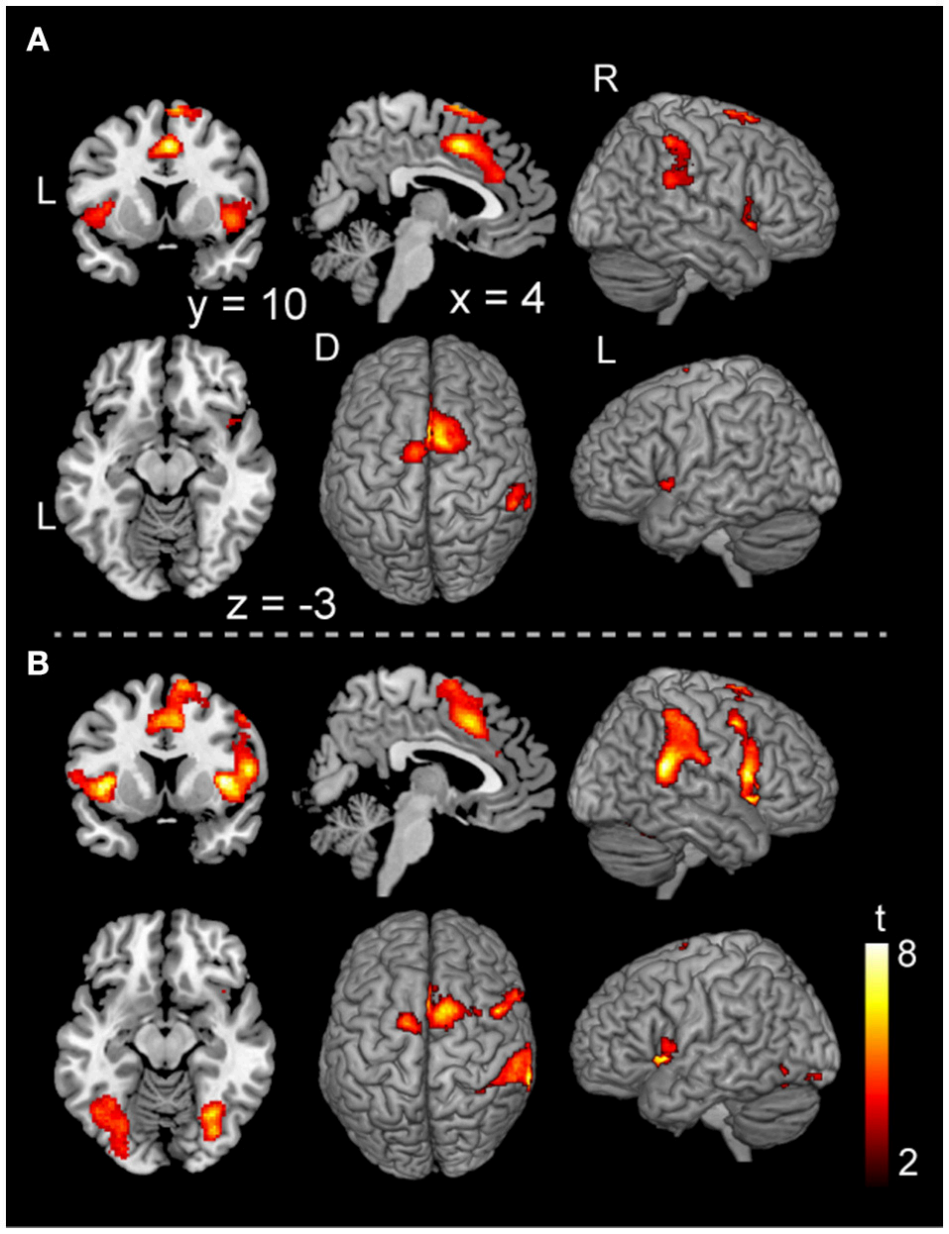
Activations of the inhibition execution in the voluntary and forced stop conditions. Activations in the voluntary stop condition **(A)** and in the forced stop condition **(B)** are shown. Activation height and extent were set thresholds at the *P* < 0.001 level (uncorrected) and a cluster-level FWE of 0.05, respectively. The scale for t-scores is shown alongside. The number and alphabetic characters indicate the XYZ coordinates in the Montreal Neurological Institute (MNI) space. The letters in the figure indicate the direction of each brain image (L, left; R, right; D, dorsal).

**Figure 5:**
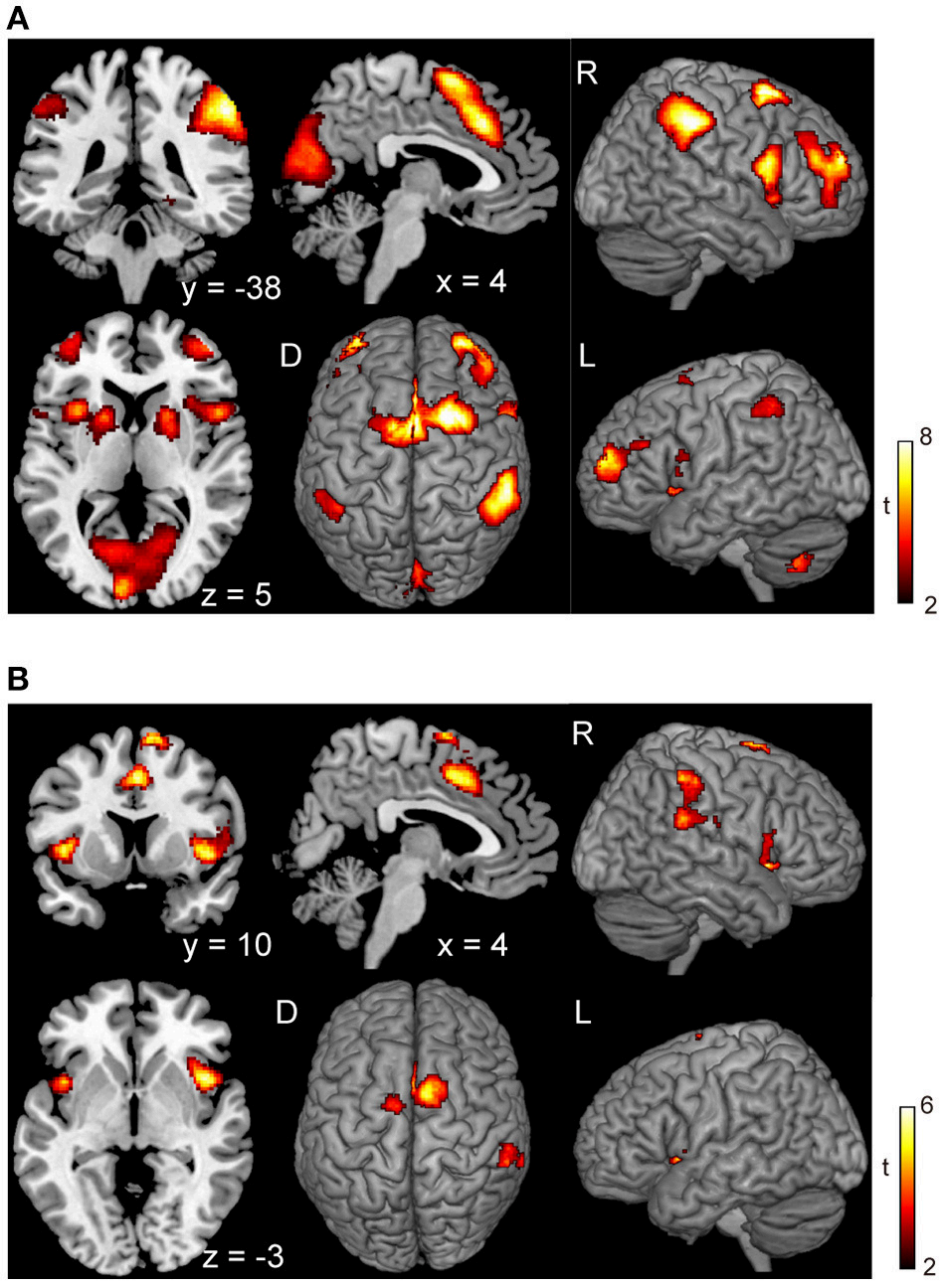
Conjunction analysis maps of the brain activations related to the planning of motor actions and the exact inhibition conduct of finger tapping. Peak activations in the conjunction analysis map for the planning of motor action between waiting condition and tapping condition **(A)** and peak activations in the map between the voluntary stop condition and forced stop condition **(B)** are shown. The height and extent thresholds of brain activation were set at the *P* < 0.001 level (uncorrected) and a cluster-level FWE of 0.05, respectively. The scale for t-scores is shown alongside. The number and alphabetic characters indicate the XYZ coordinates in the Montreal Neurological Institute (MNI) space. The letters in the figure indicate the direction of each brain image (L, left; R, right; D, dorsal).

In the voluntary stop condition, activation was found in the middle cingulate cortex (MCC), the bilateral insular cortex and the right inferior parietal lobe (rIPL) (Figure 4A and Table 4). In contrast, the forced stop condition involved brain activation in the bilateral insular cortex, the bilateral fusiform gyrus, the right angular gyrus, the MCC and the left SMA (Figure 4B and Table 4). To validate the regions of common activation, we conducted a conjunction analysis between the voluntary and forced stop conditions, revealing activation in the SMA, MCC, bilateral anterior insular cortex (AIC), right inferior parietal lobe (rIPL) and right inferior frontal gyrus (rIFG) (Figure 5B and Table 6). In this study, each stop behavior identified by an event-related regressor (indicating a behavioral change from tapping to waiting) would be to involve neural processes related to decision-making of stop actions, as well as a behavioral switch to stop the action, in both the forced and voluntary inhibition conditions.

**Table 4:** Positive activation peaks for the behavioral changes from tapping to waiting.

**Conditions**	**Regions**	**Z-value**	**MNI coordinates x,y,z**			**Number of voxels**
Voluntary stop	Middle cingulate cortex	5.31	2	10	44	1,022
	Right SMA	4.96	8	2	70	563
	Right insula	4.42	42	8	−2	575
	Right inferior parietal lobe	4.23	56	−38	52	323
	Left insula	4.19	−42	6	0	318
Forced stop	Right insula	5.70	38	10	6	1,614
	Right fusiform gyrus	5.52	38	−54	−18	1,076
	Right angular gyrus	5.51	62	−42	18	1,697
	Left insula	5.43	−36	20	6	689
	Middle cingulate cortex	5.18	8	14	44	1,648
	Left fusiform gyrus	4.86	−40	−56	−16	716
	Left SMA	4.49	−14	0	68	261

*All clusters survived the peak threshold of p < 0.001 level (uncorrected) and a cluster-level FWE of 0.05. SMA, supplementary motor area*.

**Table 5:** Common brain regions between inhibition and initiation of finger-tapping in the contrasts of the planning phase of voluntary against forced stop conditions by conjunction analysis.

**Regions**	**Z-scores**	**MNI coordinate, x, y, z**			**Number of voxels**
Left SMA	7.09	−8	2	66	4,759
Right premotor	6.83	20	12	62	
cortex (BA6/8)					
Left pre-SMA	6.56	−2	18	46	
Right inferior parietal lobe	6.52	56	−36	44	1,939
Right inferior frontal gyrus (BA44)	6.37	54	12	20	1,348
Right dorsolateral prefrontal cortex	6.24	28	56	22	1,763
Left insula	6.11	−34	12	2	884
Left globus pallidus/putamen	5.02	−18	10	4	
Left dorsolateral prefrontal cortex	5.96	−34	58	16	1,181
Left cerebellum	5.60	−34	−60	−34	1,153
Visual cortex (V1)	5.43	−6	−90	8	6,323
Right globus pallidus/ putamen	4.92	20	12	6	403
Left inferior parietal lobe	4.73	−46	−44	50	625
Left inferior frontal gyrus (BA44)	4.05	−54	12	10	235

*All clusters survived the peak threshold of p < 0.001 level (uncorrected) and a cluster-level FWE of 0.05. SMA, supplementary motor area; BA, Brodmann area*.

**Table 6:** Common brain regions between the voluntary and forced stop by conjunction analysis.

**Regions**	**Z-scores**	**MNI coordinate, x, y, z**			**Number of voxels**
Right SMA	5.42	8	2	70	1,144
Middle cingulate cortex	5.00	2	8	46	
Right insula	5.01	42	12	−2	703
Right inferior frontal gyrus (BA44)	4.18	54	16	12	
Left insula	4.78	−42	10	0	290
Right inferior parietal lobe	4.50	58	−30	48	640

*All clusters survived the peak threshold of p < 0.001 level (uncorrected) and a cluster-level FWE of 0.05. SMA, supplementary motor area; BA, Brodmann area*.

## Discussion

In the current study, the neural correlates of inhibition of an ongoing behavior were investigated regarding similarities in brain activation in planning and executing to inhibit continuing movements. Behaviorally, in the voluntary conditions, the waiting period before starting the finger-tapping action was shorter than the exercise period before stopping finger-tapping, despite the instruction being the same (Figure 1C). The difference in duration between these conditions may have occurred because the task in the start condition required self-control to suspend an action during the waiting period. Previous studies have suggested that self-control processing is intrinsically costly and aversive in terms of cognitive demand (Kool et al., 2010, 2013). The shorter duration might reflect the avoidance of high cognitive demand to maintain self-control. Meanwhile, the number of taps per second in the stop conditions was lower than that in the start conditions (Figure 1D). The different behavior between the start and stop conditions may reflect the required contradictory behavior in the stop conditions in which subjects simultaneously had to take an action and be ready to stop it. Previous studies using the go/no-go task reported slower reaction times to the visual stimuli compared with simple reaction times, because of the anticipation of the no-go signal (Liddle et al., 2001; Kida et al., 2005). In addition, our result may be partly interpreted by a task set issue because parallel processing or competitions and interactions between task rules, task items and responses reportedly occur at the single-neurons and across multiple regions during preparation and execution of a task (Sakai, 2008).

The results of the execution phase revealed activation in the MCC, including the pre-SMA, SMA, IPL, IFG, and insula cortex, in the both voluntary and forced conditions (Figure 4 and Table 4). Previous studies have reported that the SMA and pre-SMA are frequently recruited in response inhibition tasks (Mostofsky et al., 2003; Li et al., 2006; Simmonds et al., 2008). The process of response inhibition has also been reported to evoke activation in the insular cortex (Konishi et al., 1998; Aron et al., 2004; Kelly et al., 2004; Garavan et al., 2006; Brass and Haggard, 2007), and the IPL (Menon et al., 2001; Rubia et al., 2001). Moreover, other studies have reported that the right inferior frontal gyrus (rIFG) plays a crucial role in response inhibition to control impulsivity (Aron et al., 2004; Forstmann et al., 2008; Bari and Robbins, 2013). These findings are in accord with the current hypothesis that the neural correlates of inhibition of an ongoing action would be consistent with brain regions related to response inhibition.

The comparison of the execution phase between voluntary and forced conditions revealed brain activation specific to stimulus-triggered movements (stimulus-triggered > self-paced) in occipital regions and motor-related areas (Figure 3 and Table 3). These brain regions are associated with the visual processes of color and shape (Le et al., 1998) and hand movements (Weinrich and Wise, 1982; Donoghue and Sanes, 1994), and may have been recruited in the forced conditions because subjects were required to respond to color changes of a plus sign indicating whether to start or stop finger-tapping. In contrast, we found no significant activation changes in the voluntary conditions compared with the forced conditions, suggesting an absence of contribution of voluntary processes in the execution phase. In accord with our finding, a previous fMRI study reported that no significant activation was observed in a contrast between self-initiated and stimulus-triggered movement (Cunnington et al., 2002). Therefore, it is very likely that the brain regions subserving at the execution phase in the voluntary condition are common with those in the forced condition. Accordingly, the conjunction analysis between voluntary and forced conditions indicated that particular brain regions are responsible for both the voluntary and forced execution process of inhibition of ongoing actions (Figure 5B). In the current study, we first assumed that externally triggered action was not voluntary but constituted passive or forced movement. However, it is difficult to distinguish between voluntary and passive actions. The outcome of a passive action comes with decision made voluntarily. So, we believe that the externally triggered action may constitute a form of “voluntary” action. Indeed, although subjects were not required to decide when to take an action in the forced condition, they were asked to make a decision to execute the action when the cues appeared. The decision-making process involves “will,” and is embedded in volitional control (Deecke, 1996). Thus, this process is thought to be involved in both voluntary and forced conditions. If so, the neural activity in the execution phase would reflect the decision-making process of execution of action. Therefore, the common regions of activation for the execution of stop (Figure 5B) might underlie the decision-making process. This may account for the absence of significant differences in activation in the direct comparison between self-initiated and stimulus-triggered conditions.

The planning phase was associated with activation in the SMA and pre-SMA, the IPL, the IFG (BA 44), the DLPFC, the insula, the left cerebellum, the primary visual cortex and the globus pallidus/putamen, irrespective of inhibition or initiation condition (Figure 2). Interestingly, the SMA, MCC, IPL, IFG and insula were also activated during the execution phase of inhibition, but activation in the DLPFC, globus pallidus/putamen, visual cortex and cerebellum were observed only in the planning phase. Regarding the brain regions exclusively involved in the planning phase, the DLPFC is considered to operate as the center of executive function in complex cognitive tasks such as making plans for the future (Gilbert and Burgess, 2008). The globus pallidus is associated with the regulation of movement through the direct and indirect pathways of the basal ganglia (Moretti and Signori, 2016; Singh-Bains et al., 2016). The striatum, including the putamen, contributes to the learning of associations between actions and rewards, selection between competing alternatives and motivational modulation of motor behavior (Liljeholm and O'Doherty, 2012). The cerebellum subserves the coordination aspects of motor control and contributes to the planning and execution of movements (Blakemore and Sirigu, 2003; Strick et al., 2009; Schmahmann, 2010). Regarding the primary visual cortex, the current results may reflect an implicit aspect of the different instruction cues between the voluntary and forced conditions. Participants were required to retain the “self” cue in visual memory in the voluntary condition because there was no signal to execute the initiation or inhibition of action.

Brass and Haggard (2007) discussed three frameworks for the investigation of intentional action: the decision about (1) which action to execute (“what”), (2) when to execute an action (“when”), (3) whether to execute an action or not (“whether”). Previous studies examining “when” decisions in the performance of freely-timed movements reported involvement of the aMCC and SMA in self-paced movements (Ball et al., 1999; Deiber et al., 1999; Jenkins et al., 2000). Furthermore, an fMRI study of self-initiated movements indicated that a “when-network” is composed of the superior SMA, insula, BA44, anterior putamen, globus pallidus and left cerebellum (Hoffstaedter et al., 2013). These brain regions are similar to the network of regions involved in the planning of inhibition of ongoing actions (Figure 2). In the current study, the planning of action was primarily related to decisions about “when” to perform an action. Therefore, the neural correlates of planning of inhibition of ongoing actions may represent a “when-network” of intentional action.

The conjunction analysis revealed several common regions of activation while planning a motor action, with or without a continuous action being performed (Figure 5A), suggesting that the neural circuits for the planning of motor actions as for execution of motor actions may be working concurrently. In daily life, people are able to perform actions while simultaneously engaging in thought, such as jumping hurdles while thinking about the number of upcoming hurdles. Performance of dual or multitasks often requires the recruitment of different functional systems for cognitive processes and motor actions which may exhibit interference with one another, as suggested by previous studies using dual-task paradigms (Plummer et al., 2013; Carmela et al., 2017). However, the current results revealed that planning a movement was associated with a similar pattern of activation whether or not a continuous action was being performed at the same time (Figures 2, **5A**). This finding suggests that a sequence of simple motor actions generates minimal interference with the process of planning a next action.

The current finding of brain regions specific to motor inhibition working commonly during execution and planning may be extrapolated to the pathological symptom named as perseveration seen in disorders of inhibition. The functional breakdown of the brain areas found in the present analysis may be responsible for this perseveration in brain disorders (Gandola et al., 2013; Henry et al., 2013). Indeed, a set of areas found in the present study may reportedly relate to different types of perseveration (Gandola et al., 2013). The present finding, therefore, helps understand the pathophysiology of disinhibition in brain disorders.

### Limitation

In the present study, we used colored visual stimuli to instruct subjects to execute stopping or starting finger-tapping. Especially, the use of visual stimuli such as a stop or start sign in the involuntary conditions compared with the voluntary conditions may be an influencing factor to the results. To clarify this, further study will be required; for example, auditory stimuli instead of visual stimuli would be available to verify the brain regions that related to execution of stopping. In addition, the results showed the laterality of brain activations toward the right hemisphere in both the planning and execution phase (Figures 2, 4). It is likely that this tendency might be based on the use of right-hand finger tapping because of laterality of innervation of hands. On the other hand, the right IFG is known as a main region of response inhibition in the prefrontal cortex (Menon et al., 2001; Aron et al., 2004; Bari and Robbins, 2013), suggesting that the right hemisphere may be dominant for inhibition of motor control even if a task of left finger-tapping was conducted. Further studies will be needed to address whether the contralateral activation of the motor and pre-motor cortex would occur when using a left finger tapping.

## Conclusion

To verify the neuronal processes underlying the inhibition of an ongoing action, we conducted an fMRI study using a finger-tapping task involving self-initiated and stimulus-triggered inhibition of an ongoing action. We attempted to dissociate the inhibition process into planning and execution phases. In the execution phase, inhibition of ongoing action activated the SMA, aMCC, insula cortex, rIPL and rIFG, compatible with the brain activation seen during response inhibition. In addition, activation of the DLPFC, globus pallidus/putamen, premotor cortex, primary visual cortex and cerebellum were observed in the planning phase in both the initiation and inhibition conditions. These results suggest that a common inhibitory mechanism for canceling a motor action is used in both execution and planning of motor action, and that a common planning network of motor action is recruited even when an action is ongoing.

## Author Contributions

KO designed and performed the research, analyzed the data, and wrote the paper. SI and YT performed the research. YO designed the research and wrote the paper.

### Conflict of Interest Statement

YT was a predoctoral researcher, employed by company Hamamatsu Photonics KK. The remaining authors declare that the research was conducted in the absence of any commercial or financial relationships that could be construed as a potential conflict of interest.
