# Corrigendum: Similar Neural Correlates of Planning and Execution to Inhibit Continuing Actions

**DOI:** 10.3389/fnins.2018.01024

**Published:** 2019-01-11

**Authors:** Kei Omata, Shigeru Ito, Youhei Takata, Yasuomi Ouchi

**Affiliations:** ^1^Department of Biofunctional Imaging, Medical Photonics Research Center, Hamamatsu University School of Medicine, Hamamatsu, Japan; ^2^Hamamatsu Medical Photonics Foundation, Hamamatsu PET Imaging Center, Hamamatsu, Japan; ^3^Hamamatsu Photonics KK, Global Strategic Challenge Center, Hamamatsu, Japan

**Keywords:** behavioral inhibition, simple finger tapping, voluntary decision, cued judgment, neural substrates, functional magnetic resonance imaging

In the original article, there was a mistake in the legend for Figure 2 as published. The term “waiting” in Figure 2 (A) was swapped with the term “tapping” in Figure 2 (B). The correct legend appears below.

“Activations in the planning phase of an ongoing action task while tapping (A) and while waiting (B) are shown.”

The authors apologize for this error and state that this does not change the scientific conclusions of the article in any way. The original article has been updated.

